# Combination treatment of docetaxel with caffeic acid phenethyl ester suppresses the survival and the proliferation of docetaxel-resistant prostate cancer cells via induction of apoptosis and metabolism interference

**DOI:** 10.1186/s12929-022-00797-z

**Published:** 2022-02-23

**Authors:** Yu-Ke Fu, Bi-Juan Wang, Jen-Chih Tseng, Shih-Han Huang, Ching-Yu Lin, Ying-Yu Kuo, Tzyh-Chyuan Hour, Chih-Pin Chuu

**Affiliations:** 1 Institute of Cellular and System Medicine, National Health Research Institutes, No. 35, Keyan Road, Zhunan Town, 35053 Miaoli County Taiwan; 2 Department of Biochemistry, Kaohsiung Medical University, Kaohsiung, Taiwan; 3 Graduate Program for Aging and Graduate Institute of Basic Research Sciences, China Medical University, Taichung, Taiwan; 4 Biotechnology Center, National Chung Hsing University, Taichung City, Taiwan; 5 Department of Life Sciences, National Central University, Taoyuan City, Taiwan

**Keywords:** Prostate cancer, Docetaxel, Caffeic acid phenethyl ester, Apoptosis, Bcl-2, DHCR24

## Abstract

**Background:**

Docetaxel has been approved by USFDA as a first-line treatment for castration-resistant prostate cancer (CRPC) patients. Patients receiving androgen deprivation therapy along with docetaxel result in superior survival, lower serum prostate specific antigen (PSA) level, and better quality of life. However, a significant proportion of these patients ultimately develop resistance to docetaxel within months. Caffeic acid phenethyl ester (CAPE), one of the main bioactive components extracted from the propolis, has been reported to be effective for repressing the tumor growth, the migration and invasion of prostate cancer (PCa) cells, as well as the downstream signaling and stability of androgen receptor (AR). We hence determined if combination treatment of docetaxel with CAPE can suppress the proliferation and the survival of docetaxel-resistant PCa cells.

**Methods:**

We established docetaxel-resistant PC/DX25 and DU/DX50 CRPC cell lines from PC-3 and DU-145 human PCa cells, respectively. Proliferation assay, MTT assay, flow cytometry with Annexin V staining, Comet Assay, and nude mice xenograft model were applied to determine the effects of combination treatment on cell proliferation and survival of the docetaxel-resistant PCa cells. Micro-Western Array (MWA) and qRT-PCR were used to investigate the molecular mechanism lying underneath.

**Results:**

Combination treatment effectively suppressed the proliferation, survival and tumor growth of docetaxel-resistant PCa cells both in vitro and in nude mice. Comet assay and flow cytometry indicated that combination treatment induced apoptosis in docetaxel-resistant PCa cells. MWA and Western blotting assay revealed that combination treatment suppressed protein expression of Bcl-2, AKT2, c-Myc, apoptosis and caspase activation inhibitor (AVEN), pyruvate kinase M2 (PKM2) but increased protein expression of Bax, caspase 3, cytochrome c, glucose-6-phosphate dehydrogenase (G6PD) and acylglycerol kinase (AGK). Overexpression of Bcl-2 in the docetaxel-resistant PCa cells enhanced cell proliferation of docetaxel-resistant PCa cells under combination treatment. Analysis with qRT-PCR suggested that combination treatment decreased cholesterol biosynthesis genes DHCR24 (24-dehydrocholesterol reductase) and LSS (lanosterol synthase) but increased genes involved in glycolysis and TCA cycle.

**Conclusions:**

Combination treatment of docetaxel with CAPE effectively suppressed the proliferation and survival of docetaxel-resistant PCa cells via inhibition of Bcl-2 and c-Myc as well as induction of metabolism interference. Combination treatment can be beneficial for patients with docetaxel-resistant PCa.

**Supplementary Information:**

The online version contains supplementary material available at 10.1186/s12929-022-00797-z.

## Background

Androgen deprivation therapy (ADT) is the primary treatment for metastatic prostate cancer (PCa). However, most PCa patients receiving ADT develop recurrent castration-resistant prostate cancer (CRPC) within 3 years, with a median overall survival time of 1–2 years [1, 2]. Docetaxel is a commonly used cancer chemotherapy drug which belongs to the taxane class. Docetaxel is a synthetic analogue of paclitaxel, an extract from the bark of the rare *Taxus brevifolia* [3]. Docetaxel binds to the β-tubulin subunit of the microtubules, stabilizes the microtubule assembly, and prevents the depolymerization and disassembly of microtubule [4]. As a result, docetaxel reduces free tubulin in cytoplasm, inhibits the mitotic cell division, and suppresses the proliferation and survival of cancer cells [4]. Treatment with docetaxel plus prednisone has been approved by USFDA as a first-line treatment for CRPC patients. Patients with metastatic hormone-sensitive PCa receiving ADT plus docetaxel had a medium overall survival of 57.6 months, which was 13.6 months longer than that of patients receiving ADT alone [5]. Treatment with docetaxel and prednisone or docetaxel and estramustine for metastatic CRPC patients results in superior survival, improved rates of response in terms of pain, lower serum PSA level, and better quality of life as compared to treatment with mitoxantrone plus prednisone [6, 7]. However, a significant proportion of PCa patients receiving docetaxel-based therapy ultimately develop drug resistance [8, 9]. Several mechanisms have been reported to contribute to the development of docetaxel-resistance, including mutations in microtubule, evolution of cancer stem cells, elevation of Multi-Drug Resistance (MDR) family of efflux transporters, re-activation of androgen receptor (AR) signaling, upregulation of PI3K-AKT signaling [8], as well as dysregulation of PPARα (peroxisome proliferator-activated receptor α) signaling and CDH1 gene [10].

Caffeic acid phenethyl ester (CAPE) is a lipophilic derivative of caffeic acid and a phenolic antioxidant structurally related to 3,4-dihydroxycinnamic acid. CAPE is one of the main bioactive components and a strong antioxidant extracted from honeybee propolis [11, 12]. We previously reported that CAPE treatment suppressed the cell proliferation of both androgen-dependent and androgen-independent PCa cells via inhibition of AKT signaling and c-Myc [13–15]. We also discovered that CAPE treatment suppressed the phosphorylation of Ser81 and Ser213 on AR, therefore decreased the stability of AR and induced degradation of AR protein in PCa cells [16]. As re-activation of AR signaling and upregulation of AKT signaling have been reported to promote the development of resistance to docetaxel in CRPC, we investigated if combination treatment of docetaxel and CAPE can suppress the proliferation and survival of docetaxel-resistance PCa cells.

## Materials and methods

### Chemicals

Caffeic acid phenethyl ester (CAPE) was purchased from Sigma-Aldrich (St. Louis, MO, U.S.A.), while docetaxel was purchased from Cayman Chemicals (Ann Arbor, MI, U.S.A.).

### Cell culture

PC-3 and DU-145 cells were purchased from ATCC. Docetaxel-resistant sublines PC/DX25 and DU/DX50 sublines were developed by chronically exposing PC-3 and DU-145 cells to progressively increased concentrations of docetaxel. PC-3 and DU-145 cells were maintained in RPMI-1640 medium containing 10% FBS, penicillin (100 U/ml), and streptomycin (100 μg/ml) at 37 °C with 5% CO_2_. PC/DX and DU/DX cells were maintained with 25 nM and 50 nM docetaxel, respectively.

### Cell proliferation assay

PC/DX25 and DU/DX50 cells were seeded at a density of 3 × 10^3^ cells/well in 96-well plates with 100 μl RPMI-1640 medium containing 10% FBS and increasing concentration of docetaxel and CAPE for 96 h. Relative cell number was analyzed by measuring the DNA content of cell lysates with Hoechst dye 33,258-based 96-well proliferation assay (Sigma, St. Louis, MO, USA) as described previously [17]. All readouts were normalized to the average of the control condition in each individual experiment. The experiment was repeated at least eight times.

### Cell viability assay

PC/DX25 and DU/DX50 cells were seeded at a density of 5 × 10^3^ cells per well in a 96-well plate (BD Bioscience). After 24 h, the cells were treated with increasing concentrations of docetaxel and CAPE for 96 h. Cell viability was assessed by an MTT (3,4,5-dimethylthiazol-2-yl)-2-5-diphenyltetrazolium bromide) assay as previously described [15]. The amount of formazan was determined by measuring the absorbance at 560 nm using an Tecan GENios™ plate reader (Tecan group Ltd, Männedorf, Switzerland). All results were normalized to the average of the control condition in each individual experiment. All experiments were repeated at least three times.

### Comet assay

PC/DX25 and DU/DX50 cells were seeded overnight in a 6 cm dish with a density of 1 × 10^5^ cells per well in 5 ml of culture medium. On the second day, cells were treated with docetaxel (25 nM for PC/DX25 and 50 nM for DU/DX50) and increasing concentration of CAPE for additional 48 h. Comet assay was performed with OxiSelect™ Comet Assay Kit (Cell Biolabs, San Diego, CA, USA) according to the manufacturer ‘s instruction. All experiments were repeated three times.

### Xenograft experiment in nude mice

The animal protocol was reviewed and approved by the Institutional Animal Care and Use Committee (IACUC) of NHRI in Taiwan (NHRI-IACUC-100105). BALB/c nude mice of 4–5 weeks old were purchased from BioLASCO (Taipei, Taiwan). PC/DX25 cells mixed with Matrigel (BD Biosciences) and were injected subcutaneously into both flanks (1 × 10^6^ cells/side) of the mice. Mice were then randomly separated into the control and the combination treatment group, each with 5 mice. There were 5 tumors developed in the control group and 4 tumors developed in the combination treatment group. The control group received docetaxel (5 mg/kg) via i.p. twice per week while the combination treatment group received docetaxel (5 mg/kg) plus CAPE (10 mg/kg) via i.p. injection twice per week. Tumor growth was monitored by measuring the length and width of the tumor by absolute digimatic caliper. The tumor sizes were calculated using the following formula = length × width × height × 0.52. Body weight and tumor size of the mice were monitored three times a week.

### Flow cytometry

PC/DX25 cells were seeded in a 6 well-plate with a density of 2 × 10^5^ cells per well in 5 ml of culture medium for 24 h. Cells were then treated with 25 nM docetaxel and increasing concentrations of CAPE for 48 h. Flow cytometry was performed as previously described [18]. All experiments were repeated three times.

### Western blotting analysis and Micro-Western Array

Western blotting assay was performed as previously described [16]. All the antibodies used in present study were listed in Additional file 1: Table S1. Anti-rabbit and anti-mouse IgG secondary antibodies purchased from Santa Cruz Biotechnology. Intensity of bands for different proteins were quantified with Image J software after EPSON stylus TX130 scanning. Micro-Western Array (MWA) were performed as previously described [13]. Blots were analyzed by Odyssey analysis software (Li-Cor Biosciences, USA). Heatmaps were created by using PermutMatrix software (LIRMM). Antibody information was listed in Additional file 1: Table S1.

### Immunohistochemistry

Paraffin embedded tissue sections were derived from xenografts in nude mice mentioned above. The immunohistochemistry staining was performed as previously described [19]. All of antibodies used were listed in Additional file 1: Table S1.

### Data analysis

Data are presented as the mean ± SD of at least three experiments. Student’s T test (two-tailed, unpaired) was used to evaluate the statistical significance of results from proliferation assay experiments.

## Results

### Combination treatment of docetaxel with CAPE suppresses the proliferation and the survival of docetaxel-resistant PCa cell lines

**Fig. 1:**
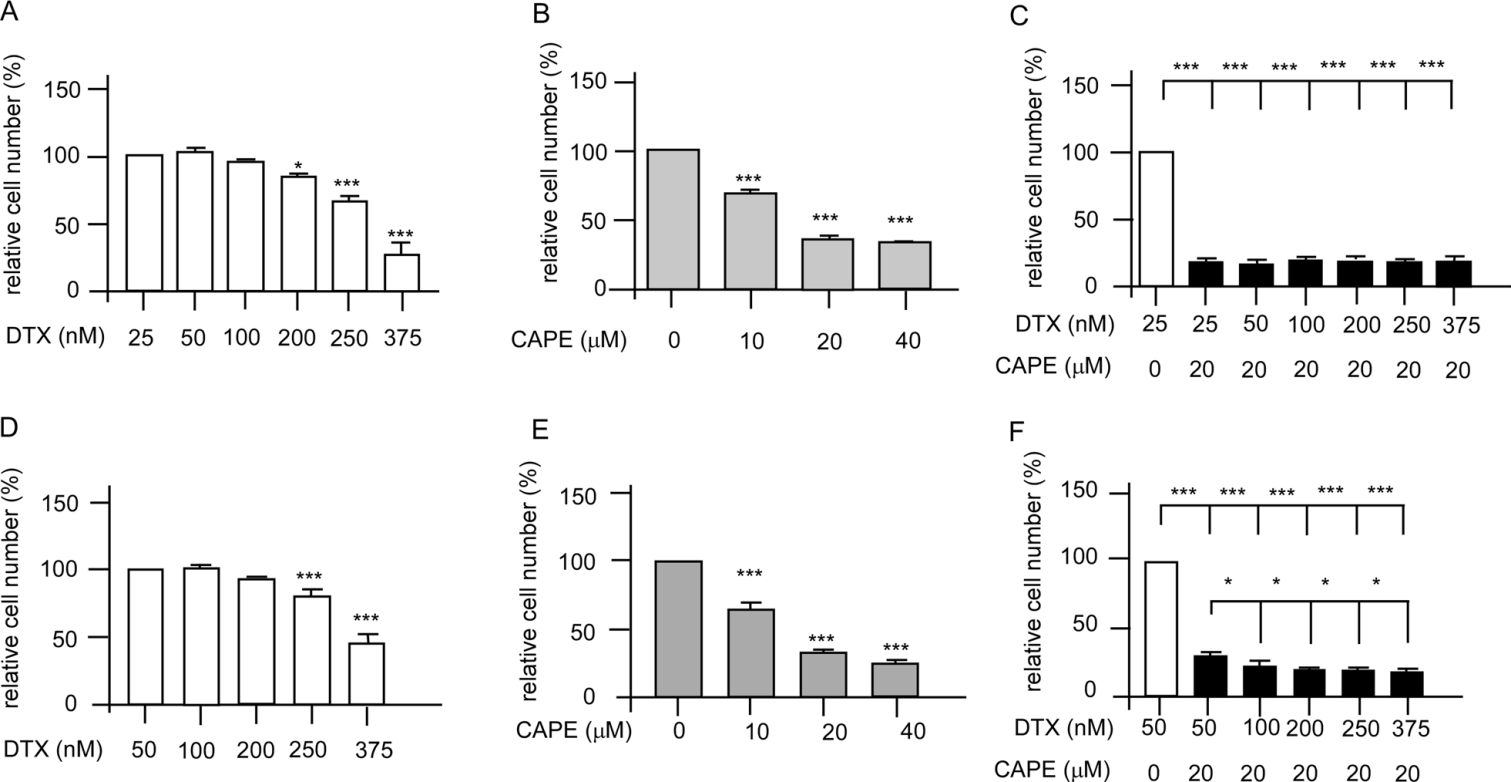
Effects of docetaxel, CAPE, and combination treatment on the proliferation of docetaxel-resistant PC/DX25 and DU/DX50 cells. The cell proliferation was determined by Hoechst 33258-based 96 well proliferation assay. PC/DX25 cells were treated with increasing concentration (25, 50, 100, 200, 250, 375 nM) of docetaxel (**A**), increasing concentration (0, 10, 20, 40 μM) of CAPE (**B**), or combination of docetaxel plus 20 μM CAPE (**C**) for 96 h. DU/DX50 cells were treated with increasing concentration (50, 100, 200, 250, 375 nM) of docetaxel (**D**), increasing concentration (0, 10, 20, 40 μM) of CAPE (**E**), or combination of docetaxel plus 20 μM CAPE (**F**) for 96 h. Results were presented as mean ± standard error. Experiments were repeated for at least three times. The asterisks * and *** represent *p* value < 0.05 and *p* value < 0.0001, respectively

Docetaxel-resistant PC/DX25 and DU/DX50 cell lines were developed by chronically exposing PC-3 and DU-145 cells to progressively increased concentrations of docetaxel. The initial concentration of docetaxel being added to the culture medium was 5 nM for both PC-3 and DU-145 cells. The survived cells were then gradually being treated wth increasing concentration of docetaxel. The final concentration of docetaxel in culture medium for maintaining the docetaxel-resistant PC-3 and DU-145 cells were 25 nM and 50 nM docetaxel, respectively. These drug-resistant PCa cells were named as PC/DX25 and DU/DX50, respectively. Hoechst dye 33,258-based proliferation assay and MTT assay were used to examine the effects of docetaxel and CAPE on cell proliferation and survival of PC/DX25 cells and DU/DX50 cells. PC/DX25 cells were treated with increasing concentration of docetaxel (25–250 nM) (Fig. 1A) or CAPE (0–40 μM) (Fig. 1B) for 96 h. IC_50_ of docetaxel and CAPE to suppress PC/DX25 cells is 316.4 nM and 15.7 μM, respectively. We then examined the effects of combination treatment of docetaxel with CAPE on proliferation of PC/DX25 cells. We chose to use 20 μM CAPE, which is close to the IC_50_ of CAPE for PC/DX25 cells. When 20 μM CAPE was used in combination with docetaxel, the IC_50_ of docetaxel for suppressing the PC/DX25 cells reduced to 35.0 nM (Fig. 1C). Similarly, DU/DX50 cells were treated with increasing concentration of docetaxel (50–375 nM) (Fig. 1D) or CAPE (0–40 μM) (Fig. 1E) for 96 h. The IC_50_ of docetaxel and CAPE to suppress DU/DX50 cells is 395.3 nM and 20.2 μM, respectively. We then examined the effect of combination treatment of docetaxel with CAPE on the proliferation of DU/DX50 cells. We chose to use 20 μM CAPE, which is close to the IC_50_ of CAPE for suppressing the DU/DX50 cells. When 20 μM CAPE was used in combination with docetaxel, the IC_50_ of docetaxel to suppress the DU/DX50 cells reduced to 68.3 nM (Fig. 1F).

**Fig. 2:**
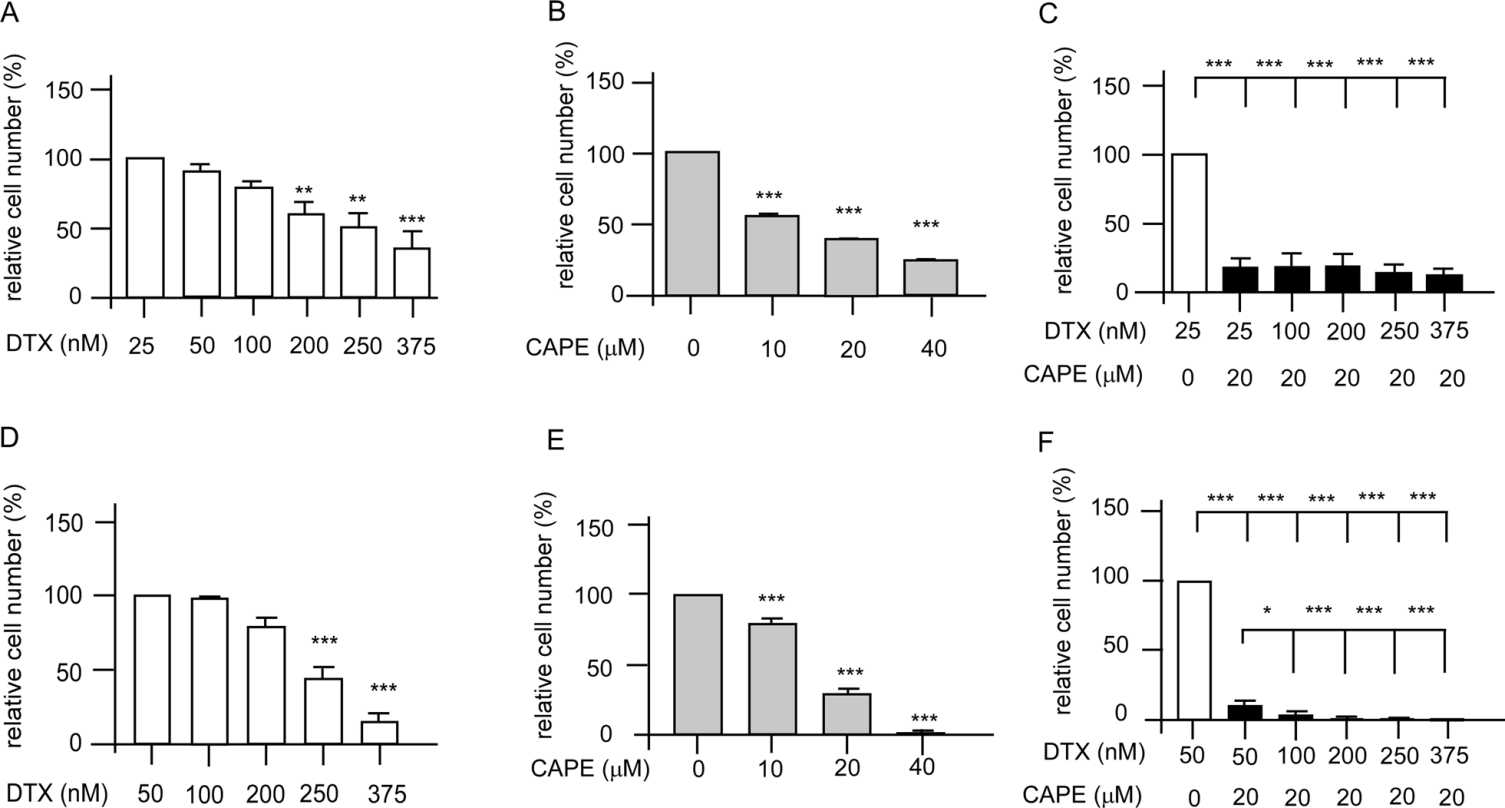
Effects of docetaxel, CAPE, and combination treatment on the survival of docetaxel-resistant PC/DX25 and DU/DX50 cells. The cell survival was determined by MTT assay. PC/DX25 cells were treated with increasing concentration (25, 50, 100, 200, 250, 375 nM) of docetaxel (**A**), increasing concentration (0, 10, 20, 40 μM) of CAPE (**B**), or combination of docetaxel plus 20 μM CAPE (**C**) for 96 h. DU/DX50 cells were treated with increasing concentration (50, 100, 200, 250, 375 nM) of docetaxel (**D**), increasing concentration (0, 10, 20, 40 μM) of CAPE (**E**), or combination of docetaxel plus 20 μM CAPE (**F**) for 96 h. Results were presented as mean ± standard error. Experiments were repeated for at least three times. The asterisks ** and *** represent *p* value < 0.01 and *p* value < 0.001, respectively

MTT assay was performed to examine the effects of combination treatment on the survival of PC/DX25 and DU/DX50 cells. The IC_50_ of docetaxel and CAPE to suppress the survival of PC/DX25 cells was 310.9 nM and 20.3 μM, respectively (Fig. 2A, B). The IC_50_ of docetaxel to suppress PC/DX25 cells reduced to 20.3 nM (Fig. 2C) when docetaxel was used in combination with 20 μM CAPE, which is close to the IC_50_ of CAPE for PC/DX25 cells. The IC_50_ of docetaxel and CAPE to suppress the survival of DU/DX50 cells is 278.0 nM and 18.5 μM, respectively (Fig. 2D, E). The IC_50_ of docetaxel to suppress the survival of DU/DX50 cells reduced to 29.7 nM (Fig. 2F) when docetaxel was used in combination with 20 μM CAPE, which is close to the IC_50_ of CAPE for DU/DX50 cells.

### Combination treatment suppresses the tumor growth of docetaxel-resistant PC/DX25 xenografts in nude mice

**Fig. 3:**
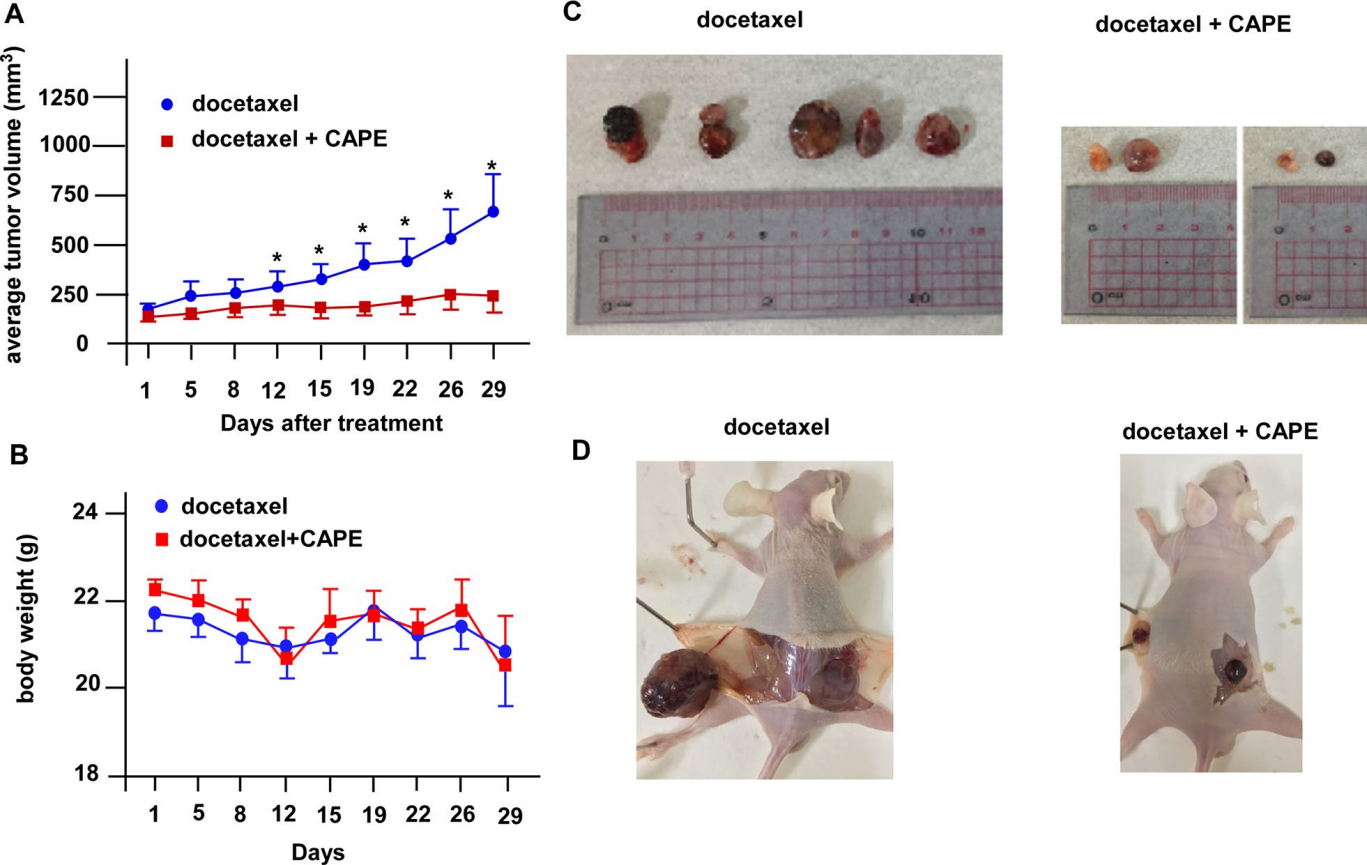
The suppressive effects of the combination treatment using docetaxel and CAPE on PC/DX25 xenografts in nude mice. **A** Docetaxel-resistant PC/DX25 cells were injected subcutaneously on both flanks (1 × 10^6^ cells/side) of the nude mice. Tumors were allowed to grow for 2 weeks to reach an average volume larger than 100 mm^3^. Mice were randomly separated into control group (receiving 5 mg/kg docetaxel twice per week via i.p. injection) or combination treatment group (receiving 5 mg/kg docetaxel plus 10 mg/kg CAPE, both twice per week via i.p. injection). Tumor growth was monitored for 29 days before mice were sacrificed. Tumors were measured twice per week with absolute digimatic calipers. Tumor volume was calculated with the formula volume = length x width x height × 0.52. **B** Body weight of mice in control group and combination treatment group was measured at the same time as tumor volume measurement. The body weight in each group was shown as mean ± standard error. **C** Photographs of xenografts in control group (docetaxel treatment only) and in combination treatment group (docetaxel plus CAPE). **D** Photographs of mice being dissected were shown for images of blood vessels nearby tumors. All data were percentage of that. Results present as mean ± standard error (n ≥ 3; **p < 0.001; ***p < 0.0001 v.s. control)

To examine if combination treatment can suppress tumor growth of docetaxel-resistant PCa cells in vivo, we inoculated PC/DX25 cells into both flanks of nude mice. The PC/DX25 xenografts continued growing in control group (receiving only docetaxel via i.p. injection, twice per week) (Fig. 3A). On the contrary, combination treatment of docetaxel plus CAPE (both via i.p. injection, twice per week) effectively suppressed the tumor growth of PC/DX25 tumors (Fig. 3A–D). Body weight of nude mice in both groups was not affected by docetaxel or CAPE treatment (Fig. 3B). During the dissecting of the tumors, we observed that combination treatment reduced the number of blood vessels around the tumors as compared to control group.

### Combination treatment induces apoptosis in the docetaxel-resistant PCa cells

**Fig. 4:**
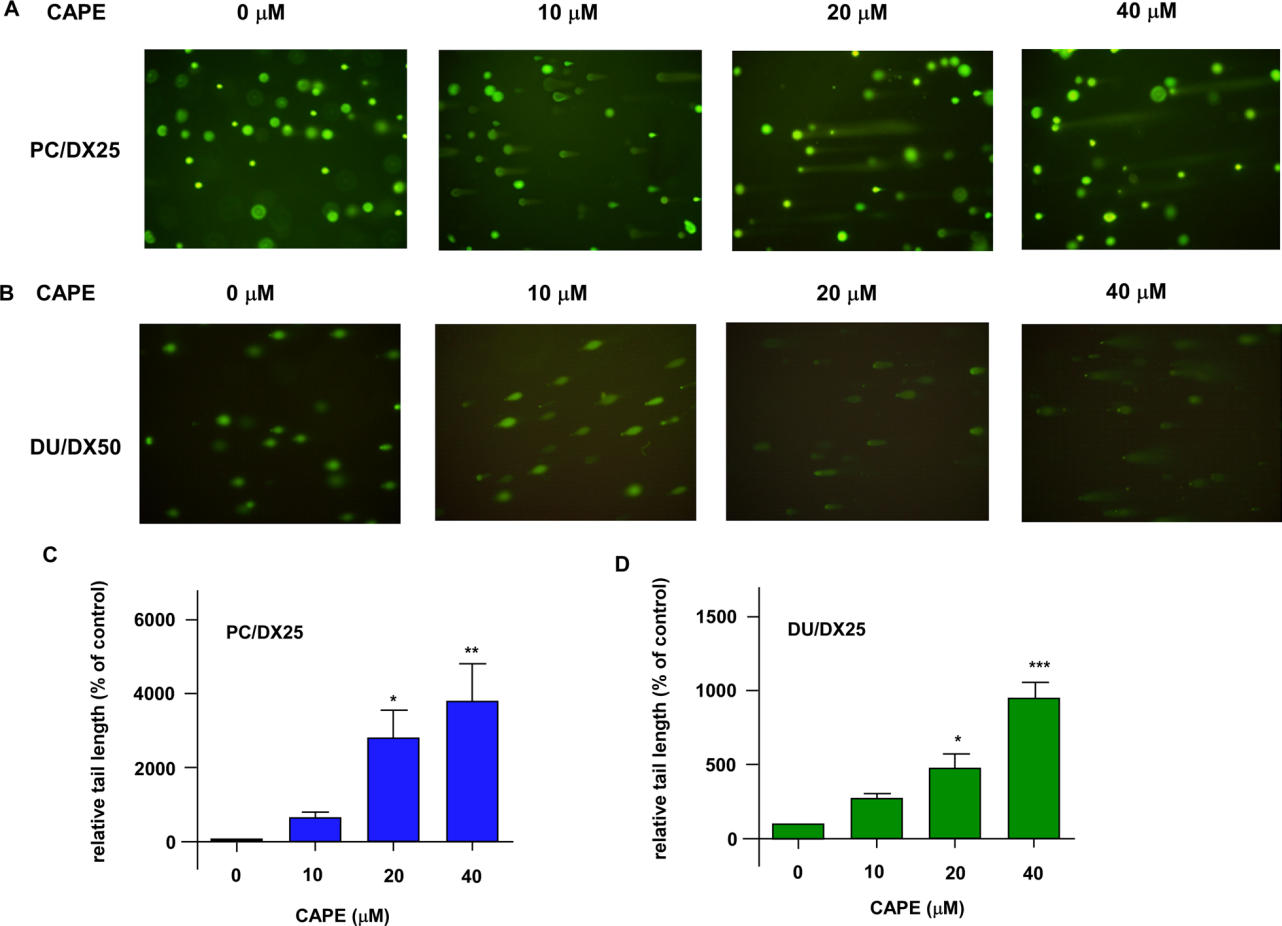
Combination treatment induced intracellular DNA damage as determined by Comet assay. Green fluorescent indicates Comet in PC/DX25 (**A**) and DU/DX50 (**B**) cells being treated with 25 nM or 50 nM docetaxel, respectively, in combination with increasing concentrations (0, 10, 20 and 40 µM) of CAPE for 96 h. Quantification of Comet tail in PC/DX25 cells (**C**) and DU/DX50 cells (**D**) was shown. The asterisks *, ** and *** represent *p* value < 0.05, *p* value < 0.01, and *p* value < 0.001, respectively

**Fig. 5:**
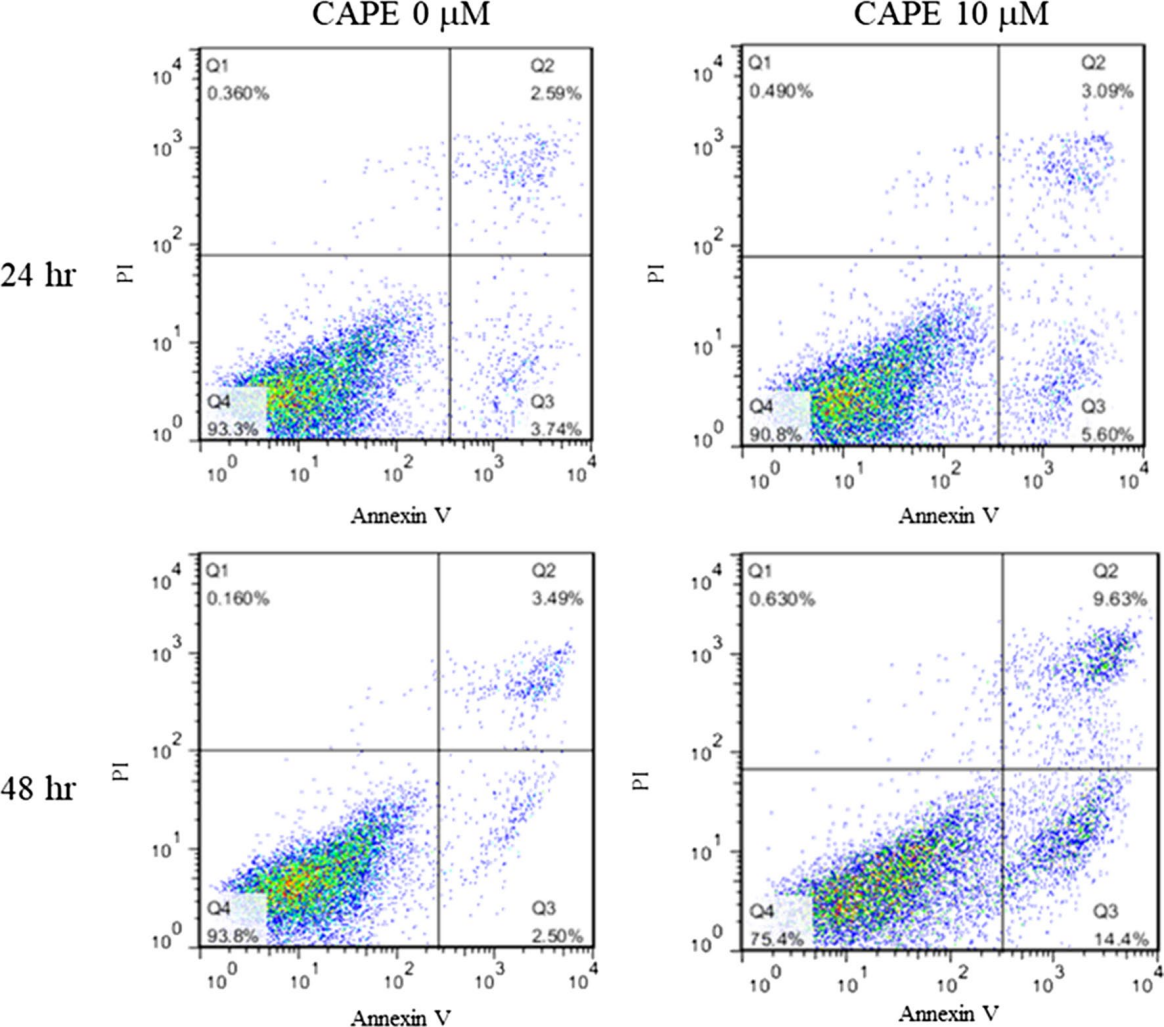
Combination treatment of docetaxel along with CAPE induced apoptosis in PC/DX25 cells as determined by Annexin V staining. PC/DX25 cells were treated with 25 nM docetaxel in combination with or without 10 µM CAPE for 24 or 48 h. Cells were harvested and stained with propidium iodide dye and Annexin V for flow cytometry analysis of apoptosis

As we observed that combination treatment decreased the survival of PC/DX25 and DU/DX50 cells, we determined if combination treatment induced apoptosis in these docetaxel-resistant PCa cells. Comet assay (Fig. 4A–D) as well as the flow cytometry analysis of Annexin V and PI (propidium iodide) staining (Fig. 5) indicated that combination treatment dose-dependently induced apoptosis in PC/DX25 and DU/DX50 cells.

### Combination treatment affects the expression of signaling proteins involved in cell survival and metabolism

**Fig. 6:**
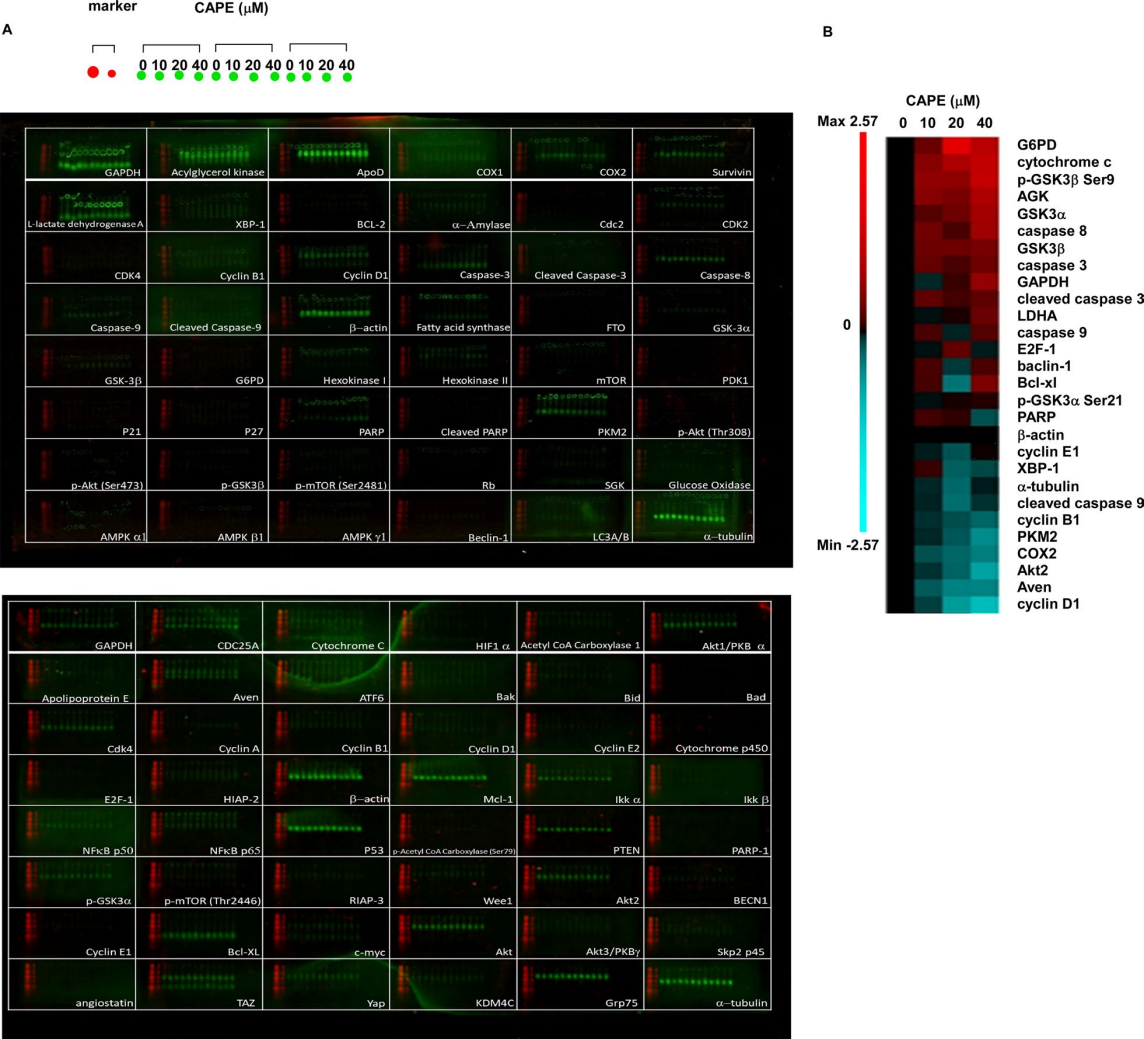
Micro-Western Array (MWA) analysis of signaling proteins affected by combination treatment in PC/DX25 cells. **A** Images of the two MWA blots analyzing the changes of expression level of signaling proteins involved in regulation of cell proliferation, survival, and metabolism in PC/DX25 cells treated with 25 nM docetaxel and increasing concentration (0, 10, 20, 40 μM) of CAPE for 96 h. Each well was loaded with two protein markers and triplicates of PC/DX25 cell samples. The names of different antibodies being used were shown at the bottom right corner of each well on the two blots. **B** Expression of proteins with changes larger than 1.5-fold increase or decrease was demonstrated with a heatmap using log_2_ value. Red color indicated an increase of protein abundance, while blue color indicated a decrease of protein abundance

To elucidate the molecular mechanisms how combination treatment suppresses the proliferation and survival of docetaxel-resistant PCa cells, we performed high-throughput Micro-Western Array (MWA) (Fig. 6A) to evaluate the expression level of 96 proteins involved in cell cycle regulation, apoptosis, AKT signaling, cholesterol efflux and metabolism. These signaling pathways are essential for the regulation of cell proliferation and survival in advanced PCa cells. Combination treatment of docetaxel and CAPE increased the protein abundance of G6PD (glucose-6-phosphate dehydrogenase), cytochrome c, phospho-GSK3β Ser9, AGK (acylglycerol kinase), GSK3α (Glycogen Synthase Kinase-3α), cleaved caspase 3 but decreased protein abundance of cyclin D1, AVEN (apoptosis and caspase activation inhibitor), AKT2, COX2, PKM2 (Pyruvate Kinase M2), and cyclin B1 (Fig. 6B).

**Fig. 7:**
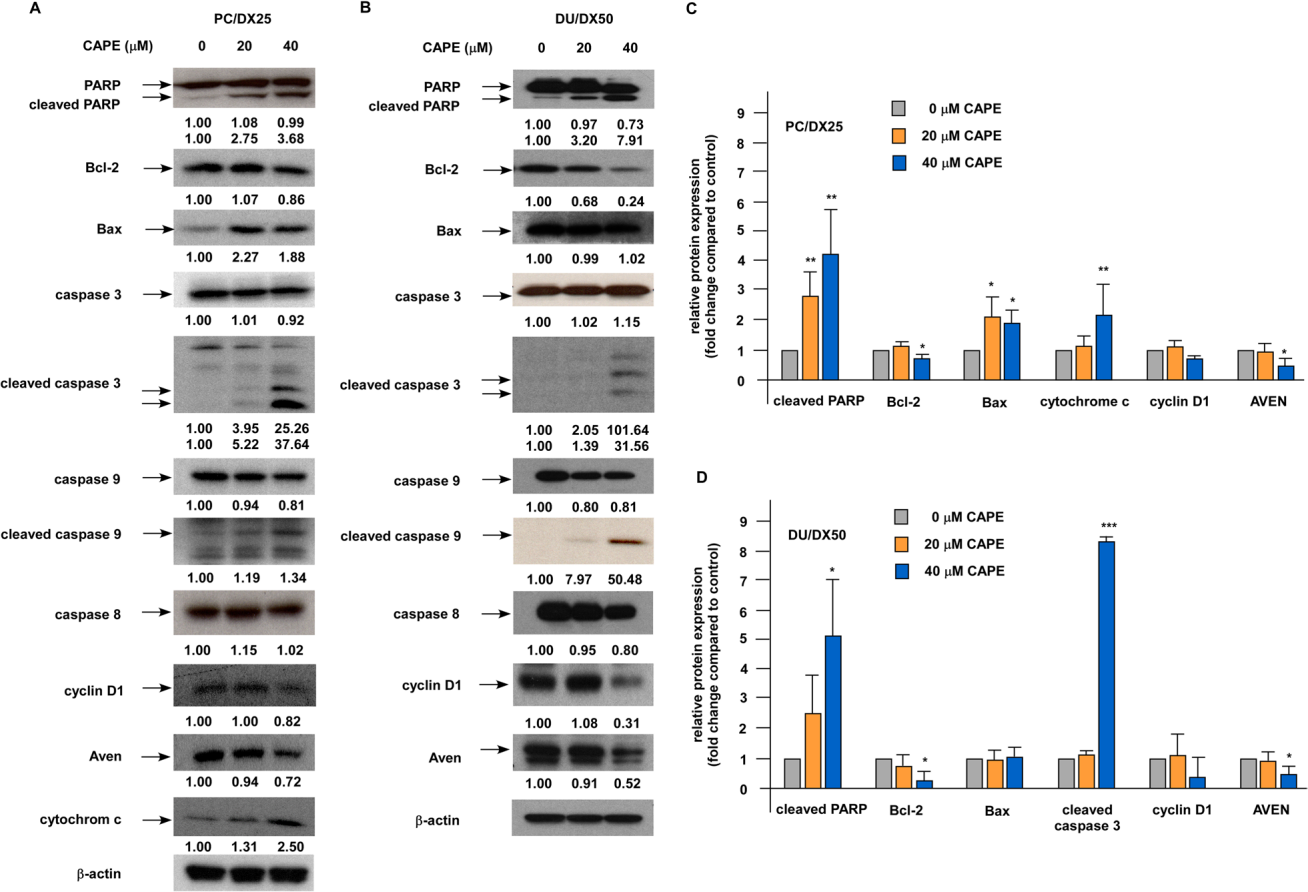
Combination treatment affected the expression of apoptosis regulatory proteins in PC/DX25 and DU/DX50 cells. PC/DX25 (**A**) and DU/DX50 (**B**) cells were treated with 25 nM or 50 nM docetaxel, respectively, in combination with 0, 20, or 40 μM CAPE for 96 h. Protein expression of PARP, Bcl-2, Bax, caspase 3, cleaved caspase 3, caspase 9, cleaved caspase 9, caspase 8, cyclin D1, AVEN, and cytochrome c was examined by Western blotting. The β-actin was used as loading control. Quantification for protein expression level of important regulatory proteins from at least three different experiments was shown for PC/DX24 (**C**) and DU/DX50 (**D**) cells

**Fig. 8:**
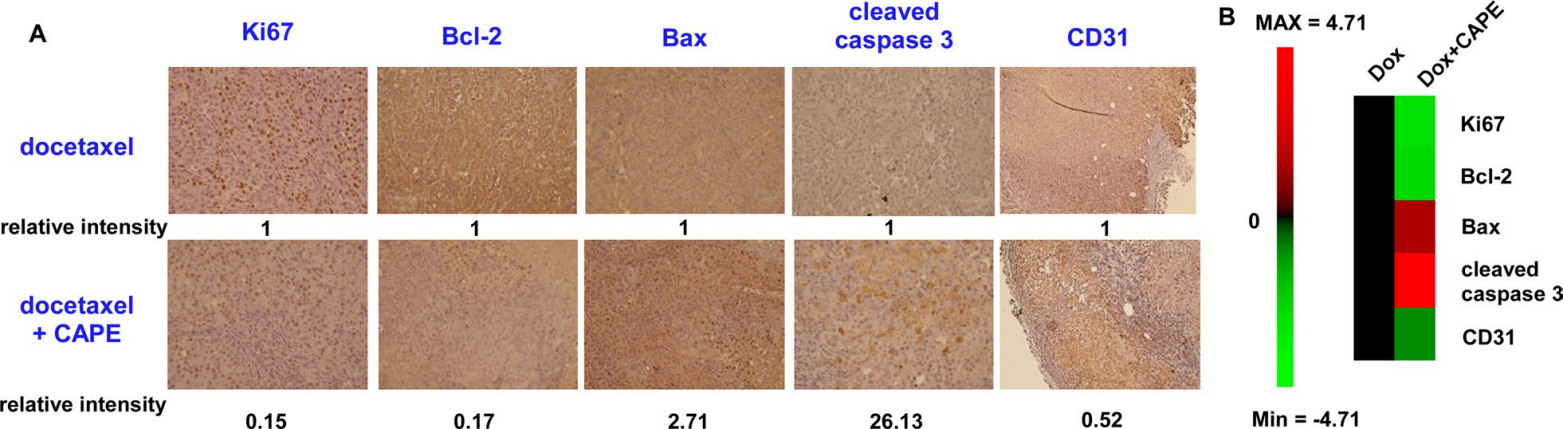
Combination treatment affected the expression of proteins regulating cell proliferation, apoptosis, and agiogenesis in PC/DX25 xenografts. **A** Protein expression level of Ki67, Bcl-2, Bax, cleaved caspase-3, and CD31 in prostate tissues of control group mice (docetaxel treatment plus control vehicle) or combination treatment group mice (docetaxel treatment plus CAPE) carrying PC/DX25 xenografts was examined by immunohistochemistry staining. The magnification for images is 400X. Relative protein expression intensity was shown under each IHC image. **B** The relative protein expression intensity for Ki67, Bcl-2, Bax, cleaved caspase-3, and CD31 in prostate tissues of control group mice vs. combination treatment group mice was shown as heatmap using log_2_ value

Western blotting revealed that combination treatment increased the protein expression level of PARP (poly ADP-ribose polymerase), cleaved PARP, Bax, cleaved caspase 3, cleaved caspase 9, and cytochrome c but decreased the protein abundance of Bcl-2, cyclin D1, and AVEN in PC/DX25 and DU/DX50 cells (Fig. 7A–D). These observations confirmed that combination treatment induced apoptotic proteins in docetaxel-resistant PCa cells. Similarly, immunohistochemistry staining revealed that combination treatment increased the protein expression level of Bax and cleaved caspase 3, but decreased that of Ki67, Bcl-2, and CD31 in PC/DX25 tumors (Fig. 8A, B).

### Overexpression of Bcl-2 increases the cell proliferation of docetaxel-resistant PCa cells under combination treatment

**Fig. 9:**
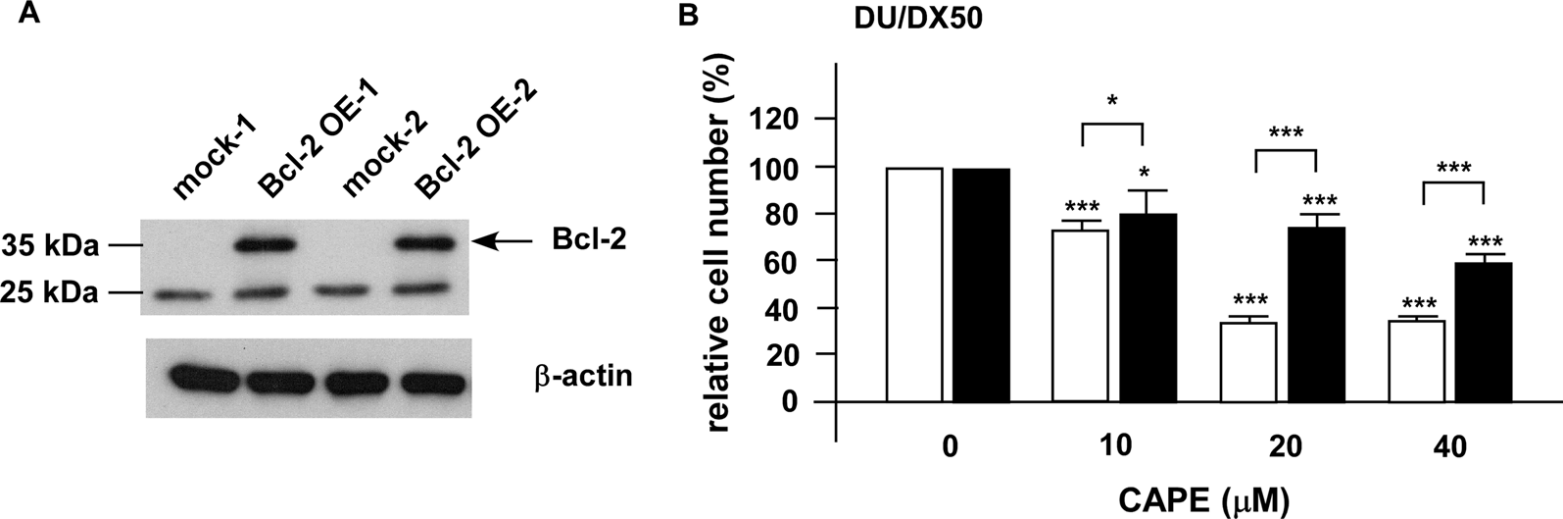
Overexpression of Bcl-2 increased cell proliferation of DU/DX50 cells under combination treatment. **A** A representative image of two clones of DU/DX50 cells transiently overexpressing Bcl-2 being confirmed by Western blotting. **B** Proliferation of control DU/DX50 and DU/DX50 cells overexpressing Bcl-2 being treated with 50 nM docetaxel plus increasing concentration of CAPE (0, 10, 20, 40 μM) for 96 h was examined by proliferation assay. Results were presented as mean ± standard error. Experiments were repeated for at least three times. The asterisks * and *** represent *p* value < 0.05 and *p* value < 0.0001, respectively

As we demonstrated that combination treatment lessened the protein abundance of Bcl-2 in PCa cells both in vitro (Figs. 6, 7) and in animal model (Fig. 8), we determined if Bcl-2 is one of the main targets for combination treatment. We used DU/DX50 cells for Bcl-2 overexpression as the combination treatment caused a more significant reduction of Bcl-2 in DU/DX50 cells (Fig. 7B) as compared to that in PC/DX25 cells (Fig. 7A). Overexpression of Bcl-2 in DU/DX50 cells (Fig. 9A) enhanced the cell proliferation of DU/DX50 cells under combination treatment (Fig. 9B). There results implied that the combination treatment suppressed the proliferation and survival of docetaxel-resistant PCa cells, at least partially, via the inhibition of Bcl-2.

### Combination treatment affects the expression of metabolic genes

**Fig. 10:**
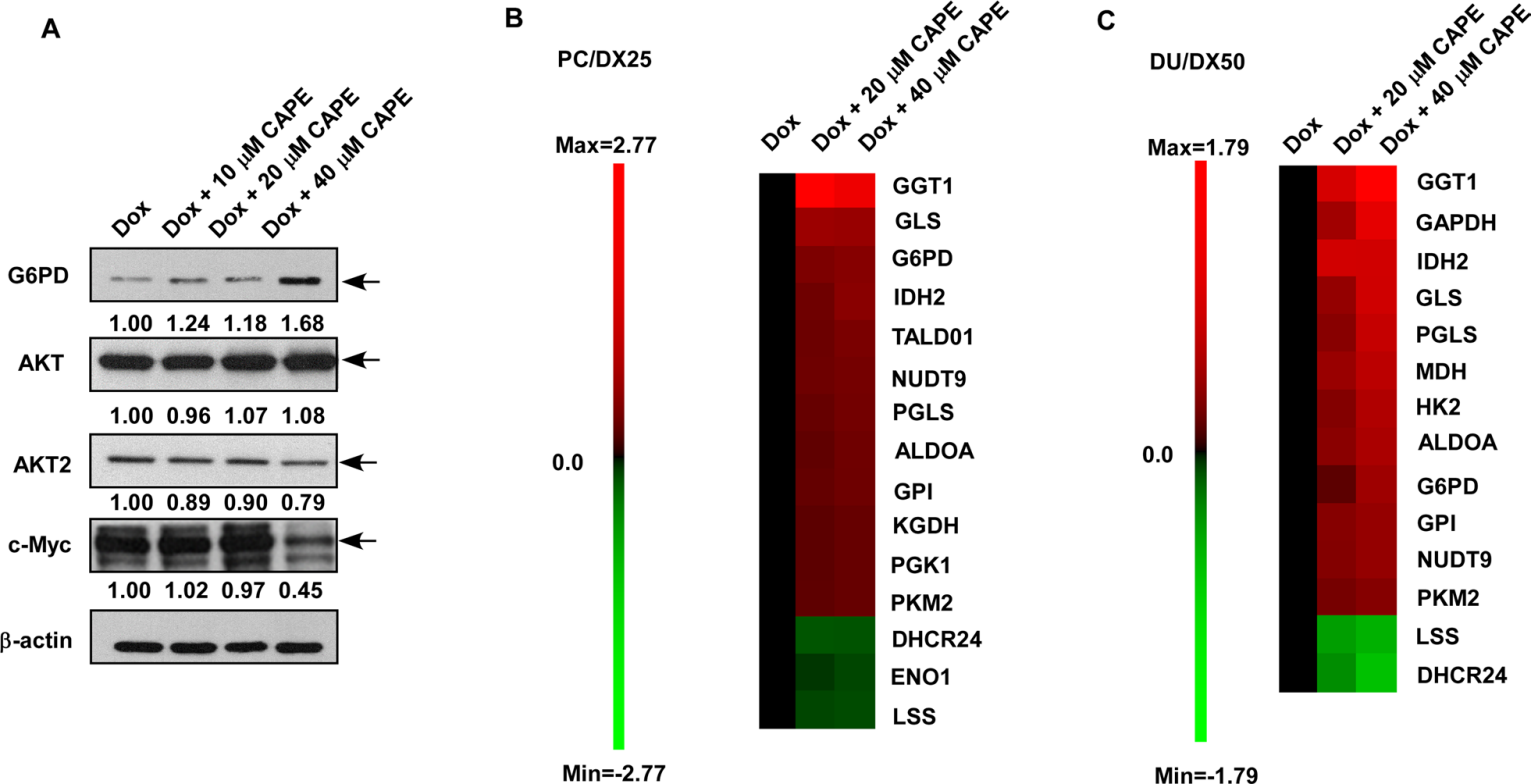
Combination treatment affected expression of proteins and genes involved in metabolism regulation. **A** Effects of combination treatment on protein expression of G6PD, AKT, AKT2, and c-Myc in PC/DX25 cells treated with 25 nM docetaxel plus increasing concentration of CAPE (0, 10, 20, 40 μM) for 96 h were examined by Western blotting. The β-actin was used as loading control. Effects of combination treatment on expression of metabolic genes in PC/DX25 cells (**B**) and DU/DX50 cells (**C**) being treated with 25 nM or 50 nM docetaxel, respectively, along with increasing concentration of CAPE (0, 10, 20, 40 μM) for 48 h were examined by qRT-PCR. Genes with change larger than 2 folds were shown by heatmap using log_2_ values

MWA analysis suggested that the combination treatment altered the expression of proteins involved in metabolism regulation (Fig. 6). Western blotting assay revealed that CAPE dose-dependently increased protein abundance of G6PD but decreased the protein expression of AKT2 and c-Myc proteins (Fig. 10A). These proteins are important in the regulation of cancer metabolism. We therefore performed qRT-PCR to examine the effects of combination treatment on metabolic genes involved in glycolysis, tricarboxylic acid cycle (TCA) cycle, glutamine metabolism, and nucleotide metabolism (Additional file 2: Fig. S1 and Additional file 3: Fig. S2). Combination treatment increased the gene expression of GGT1, GLS, G6PD, IDH2, NUTD9, TALD01, PGLS, GPI, ALDOA, PKM2, PGK1, and KGDH but decreased the gene expression of DHCR24, LSS and ENO1 in PC/DX25 cells (Fig. 10B). Similarly, the combination treatment increased the gene expression of GGT1, IDH2, GAPDH, MDH, PGLS, G6PD, GLS, ALDOA, GPI, HK2, NUDT9, and PKM2 but decreased the gene expression of DHCR24 and LSS in DU/DX50 cells (Fig. 10C). These observations indicated that the combination treatment interfered metabolism in PC/DX25 and DU/DX50 cells.

## Discussion

Docetaxel plus prednisone has been approved by USFDA as a first-line treatment for metastatic CRPC patients. Patients receiving ADT plus docetaxel have longer survival, lower serum PSA level, and better quality of life [5–7, 9]. A significant proportion of CRPC patients who initially respond to docetaxel therapy ultimately develop drug resistance [8, 9]. Therefore, novel therapeutic agents are in need for treating docetaxel-resistant PCa. In this study, we demonstrated that the combination treatment of docetaxel and CAPE can effectively suppress the proliferation and survival of docetaxel-resistant PC/DX25 and DU/DX50 PCa cells both in vitro and in nude mice xenograft model. We developed docetaxel-resistant PC/DX25 and DU/DX50 cell lines by chronically exposing PC-3 and DU-145 cells to progressively increased concentrations of docetaxel, with an initial concentration of 5 nM docetaxel to a final concentration of 25 nM and 50 nM docetaxel for PC-3 and DU-145 cells, respectively. Previous clinical study reported that patients receiving docetaxel (35 mg/m^2^) weekly via infusion showed a half-life of docetaxel clearance to be approximately 17 h, and the study demonstrated an average concentration of plasma docetaxel on day 8 after docetaxel treatment to be approximately 1.08 ± 0.51 nM [20]. According to the proliferation assay, the IC_50_ for docetaxel to suppress the proliferation of docetaxel-resistant PC/DX25 and DU/DX50 cells was 316.4 nM and 395.3 nM, respectively. In addition, PC/DX25 and DU/DX50 cells were cultured in 25 nM and 50 nM docetaxel, respectively. The concentration of docetaxel we used for establishing docetaxel-resistant PCa cell lines, 5–50 nM, reflected the concentration of docetaxel in patient serum. The drug-resistance of the PCa cell lines we developed may reflect the docetaxel-resistant prostate tumors in patients. In our combination treatment experiments, we used 20 μM CAPE for both PC/DX25 and DU/DX50 cells. The achievable concentration of CAPE in human serum is approximately 17 μM [21]. As a result, the dosage of CAPE being used in our combination treatment study is achievable in clinical treatment.

Bcl-2 is an anti-apoptotic oncoprotein. Elevation of Bcl-2 is observed in CRPC tumors [22] and up-regulation of Bcl-2 is essential for the development of CRPC [23]. It has been reported that TGF-β promotes resistance to docetaxel in PC-3 and DU-145 cells via acetylating KLF5 and inducing Bcl-2 [24], suggesting that Bcl-2 plays an essential role in the development of docetaxel resistance in PCa cells. Expression of Bcl-2 protein level in tumors is a predictive marker of outcome for CRPC patients receiving taxane-based chemotherapy [25]. Combination therapy of a phosphorothioate antisense oligonucleotide targeting Bcl-2 with docetaxel increased the overall survival of CRPC patients [26]. The subpopulation of CRPC cells which acquire docetaxel-resistance were found to lack differentiation markers and HLA class I (HLAI) antigens, while these cells exhibited elevated level of the Notch and Hedgehog signaling pathways [27]. Inhibition of Notch and Hedgehog signaling via suppression of the AKT signaling and Bcl-2 depleted the docetaxel-resistant cell population in CRPC tumors [27]. CAPE treatment has been shown to increase docetaxel and paclitaxel potency in PC-3, DU-145 and LNCaP cells [28]. In our current study, combination treatment of docetaxel and CAPE significantly reduced the protein expression of Bcl-2 in docetaxel-resistant PCa cells both in cell culture and xenograft tumors. We demonstrated that overexpression of Bcl-2 increased the cell proliferation of docetaxel-resistant PCa cells under combination treatment, suggesting that combination treatment induced apoptosis and growth inhibition in docetaxel-resistant PCa cells, at least partially, via the suppression of Bcl-2. In addition, the suppression of AKT2 and c-Myc by combination treatment (Fig. 10A) will also contribute to the inhibition of proliferation and survival of docetaxel-resistant PCa cells.

DHCR24 gene encodes the protein 3β-dehydrocholesterol-Δ24-reductase, which is a key enzyme in the cholesterol synthesis Bloch and Kandutsch-Russell pathway [29]. DHCR24 catalyzes desmosterol to cholesterol in Bloch pathway and catalyzes lanosterol to 24,25-dihydrolanosterol in the Kandutsch-Russell pathway [29]. Suppression of DHCR24-mediated cholesterol biosynthesis and lipid rafts formation results in inhibition of tumor growth and invasion of hepatocellular carcinoma [30]. DHCR24 promotes the proliferation of bladder cancer cells and bladder cancer patients have better clinical outcomes with lower DHCR24 expression in tumors [31]. Additionally, DHCR24 promotes the cell proliferation of cancer stem cells of breast cancer via regulation of Hedgehog pathway [29]. DHCR24 also aggravates the invasion and progesterone resistance in endometrial cancer cells [32]. Activation of de novo cholesterogenesis is essential for the development and progression of PCa. Suppression of cholesterol biosynthesis has been shown to reduce the survival and tumor growth of enzalutamide-resistant CRPC cells [33]. Expression of DHCR24 is higher in PCa tumors as compared to adjacent normal prostate tissues and the expression of DHCR24 is regulated by androgen in prostate tumors [34]. Lanosterol synthase (LSS) catalyzes the conversion of 2,3-oxidosqualene to lanosterol, the first sterol in the cholesterol biosynthetic pathway [35]. Inhibition of LSS suppresses the proliferation of glioma cells [36]. In our current study, the repression of DHCR24 and LSS by combination treatment in both PC/DX25 and DU/DX50 cells may interfere the cholesterol biosynthesis and therefore induce growth inhibition in these docetaxel-resistant PCa cells.

Interestingly, we observed that the combination treatment activated several genes involved in pentose phosphate pathway, tricarboxylic acid cycle (TCA) cycle, and glutamine metabolism. Glucose-6-phosphate dehydrogenase (G6PD) is the first and rate-limiting enzyme of the pentose phosphate pathway (PPP), which catalyzes the oxidation of glucose-6-phosphate and eventually produces ribose and NADPH via the pentose phosphate pathway (PPP). Both ribose and NAPDH are essential for the synthesis of cellular building materials, such as nucleic and fatty acids. G6PD is one of the major enzymes involved in cell survival following redox imbalance and it plays an essential role to protect cells from stress-induced apoptosis [37]. 6-Phosphogluconolactonase (PGLS) catalyzes the second step of the PPP pathway via hydrolyzing 6-phosphogluconolactone to 6-phosphogluconic acid [38]. Gamma-glutamyltransferase 1 (GGT1) catalyzes the transfer of the glutamyl moiety of glutathione to various amino acids [39]. Glutaminase (GLS) converts glutamine to glutamate [40]. Isocitrate dehydrogenases 2 (IDH2) catalyzes the oxidative decarboxylation of isocitrate into α-ketoglutarate in TCA cycle [41]. Acylglycerol kinase (AGK) is a multi-substrate lipid kinase which catalyzes the phosphorylation of acylglycerols to generate lysophosphatidic acid (LPA) [42]. AGK is reported to inhibit the apoptosis via activation of the NF-κB signaling in hepatocellular carcinoma [42]. It is possible that the docetaxel-resistant PCa cells try to overcome the stress of combination treatment and manage to survive via inducing a compensatory-induction of G6PD, PGLS, GGT1, GLS, IDH2, and AGK for activation of glycolysis, TCA cycle, and lipid metabolism. The fact that the docetaxel-resistant PCa cells went apoptosis 96 h after the treatment of docetaxel plus CAPE suggested that the compensatory-induction of metabolic genes cannot rescue the cancer cells.

CD31 (also known as Platelet and Endothelial Cell Adhesion Molecule 1, PECAM1) is highly expressed on the surface of endothelial cells and is a well-established marker for angiogenesis [43]. We observed that combination treatment decreased the protein abundance of CD31 in PC/DX25 xenografts and reduced the blood vessels around the tumors, suggesting that combination treatment may suppress the angiogenesis of docetaxel-resistant prostate tumors.

## Conclusions

In conclusion, our current study suggested that the combination treatment of docetaxel with CAPE can restore the sensitivity of docetaxel-resistant PCa cells to docetaxel treatment. Combined treatment of CAPE and docetaxel effectively suppressed the proliferation and survival of docetaxel-resistant PCa cells via inhibition of Bcl-2, c-Myc, AKT2 and induction of metabolism interference. Combination treatment of CAPE and docetaxel may be beneficial for PCa patient acquired docetaxel resistance.

## Supplementary Information

**Additional file 1: Table S1.** All antibodies used in Western blotting, Micro-Western blotting and IHC staining assay were listed for the name of antibody and the company information.

**Additional file 2: Figure S1.** Effects of combination treatment on metabolic genes in PC/DX25 cells. Effects of combination treatment on metabolic genes in PC/DX25 cells treated with 25 nM docetaxel plus increasing concentration of CAPE (0, 10, 20, 40 μM) for 48 h were examined by qRT-PCR.

**Additional file 3: Figure SS.** Effects of combination treatment on metabolic genes in PC/DX25 cells. Effects of combination treatment on metabolic genes in DU/DX50 cells treated with 50 nM docetaxel plus increasing concentration of CAPE (0, 10, 20, 40 μM) for 48 h were examined by qRT-PCR.

## Data Availability

The data presented in the study are included in the article and supplementary material.

## Abbreviations

AKT: Protein kinase B
ADT: Androgen deprivation therapy
AGK: Acylglycerol kinase
AR: Androgen receptor
AVEN: Apoptosis and caspase activation inhibitor
CAPE: Caffeic acid phenethyl ester
CRPC: Castration-resistant prostate cancer
DMEM: Dulbecco’s Modified Eagle Medium
FBS: Fetal bovine serum
G6PD: Glucose-6-phosphate dehydrogenase
GAPDH: Glyceraldehyde 3-phosphate dehydrogenase
GGT1: Gamma-glutamyltransferase 1
GLS: Glutaminase
GSK3α: Glycogen synthase kinase-3α
IDH2: Isocitrate dehydrogenases
LPA: Lysophosphatidic acid
LSS: Lanosterol synthase
MDR: Multi-drug resistance
MTT: 3-(4,5-Dimethylthiazol-2-yl)-2,5-diphenyltetrazolium bromide
MWA: Micro-Western array
PCa: Prostate cancer
PBS: Phosphate buffered saline
PGLS: Phosphogluconolactonase
PI: Propidium iodide
PI3K: Phosphatidylinositol-3-kinase
PKM2: Pyruvate kinase M2
PPARα: Peroxisome proliferator-activated receptor α
PPP: Pentose phosphate pathway
PSA: Prostate specific antigen
PTEN: Phosphatase and tensin homologue deleted on chromosome ten
TCA cycle: Tricarboxylic acid cycle
