# Supplementary material for: Combination treatment of docetaxel with caffeic acid phenethyl ester suppresses the survival and the proliferation of docetaxel-resistant prostate cancer cells via induction of apoptosis and metabolism interference
Source: J Biomed Sci. 2022 Feb 23;29:16. doi: 10.1186/s12929-022-00797-z (PMC8864857; doi:10.1186/s12929-022-00797-z)
Supplement: Supplementary file 1 — Additional file 1: Table S1. All antibodies used in Western blotting, Micro-Western blotting and IHC staining assay were listed for the name of antibody and the company information. [file 12929_2022_797_MOESM1_ESM.docx]

| **Antibody** | **company** |
| --- | --- |
| GAPDH | Novus |
| Acylglycerol kinase | abcam |
| ApoD | abcam |
| COX1 | abcam |
| COX2 | abcam |
| Glucose Oxidase | abcam |
| LDHA | abcam |
| XBP-1 | abcam |
| Ki67 | abcam |
| bcl-2 | BD |
| α-Amylase | Cell-signaling |
| cdc2 | Cell-signaling |
| CDK2 | Cell-signaling |
| CDK6 | Cell-signaling |
| Cyclin B1 | Cell-signaling |
| Cyclin D1 | Cell-signaling |
| Caspase-3 | Cell-signaling |
| Cleaved Caspase-3 | Cell-signaling |
| caspase-8 | Epitomics |
| Caspase-9 | Cell-signaling |
| Cleaved Caspase-9 | Cell-signaling |
| fatty acid synthase | Cell-signaling |
| FTO | Cell-signaling |
| GSK-3α | Cell-signaling |
| GSK-3β | Cell-signaling |
| G6PD | Cell-signaling |
| Hexokinase I | Cell-signaling |
| Hexokinase II | Cell-signaling |
| mTOR | Cell-signaling |
| PDK1 | Cell-signaling |
| p21 Waf1/Cip1 | Cell-signaling |
| p27(Kip1) | Cell-signaling |
| PARP | Cell-signaling |
| Cleaved PARP | Cell-signaling |
| PKM2 | Cell-signaling |
| Phospho-Akt (Thr308) | Cell-signaling |
| Phospho-Akt (Ser473) | Cell-signaling |
| phospho-GSK3β (Ser9) | Cell-signaling |
| **Antibody** | **company** |
| Phospho-mTOR (Ser2481) | Cell-signaling |
| Rb | Cell-signaling |
| SGK | Cell-signaling |
| Survivin | Cell-signaling |
| AMPK alpha 1 | Epitomics |
| beta-actin | Novus |
| AMPK beta 1 | Epitomics |
| AMPK gamma-1 | Epitomics |
| Beclin-1 | abcam |
| LC3A/B | abcam |
| CDC25A | Genetex |
| Cytochrome C | Genetex |
| HIF1 alpha | Genetex |
| Acetyl CoA Carboxylase 1 | millipore |
| Akt1/PKBα | millipore |
| Apolipoprotein E | millipore |
| Aven | Millipore |
| ATF6 | millipore |
| Bak | Millipore |
| Bid | Millipore |
| Bad | Millipore |
| Cdk4 | millipore |
| Cyclin A | millipore |
| Cyclin B1 | millipore |
| Cyclin D1 | millipore |
| Cyclin E2 | millipore |
| Cytochrome P450 | millipore |
| E2F-1 | millipore |
| HIAP-2 | Millipore |
| Mcl-1 | Millipore |
| IKKα | millipore |
| IKKβ | millipore |
| NFĸB p50 | millipore |
| NFĸB, p65 | Stanta Cruz |
| p53 | millipore |
| p-Acetyl CoA Carboxylase | millipore |
| **Antibody** | **company** |
| PTEN | millipore |
| PARP-1 (p116/p25) | millipore |
| phospho-GSK3α (Ser21) | millipore |
| phospho-mTOR (Thr2446) | millipore |
| RIAP-3 | Millipore |
| Wee1 | Millipore |
| Akt2 | Novus |
| BECN1 | Stanta Cruz |
| Cyclin E1 | Thermo |
| BCl-XL | Cell-signaling |
| c-myc | abcam |
| Akt | Cell-signaling |
| Akt3/PKBγ | millipore |
| Skp2 p45 | Stanta Cruz |
| Angiostatin | abcam |
| TAZ | Cell-signaling |
| Yap | Cell-signaling |
| KDM4C | Novus |
| Grp75 | Cell-signaling |
| alpha-tubulin | Genetex |
