# Supplementary figures and images for: Combination treatment of docetaxel with caffeic acid phenethyl ester suppresses the survival and the proliferation of docetaxel-resistant prostate cancer cells via induction of apoptosis and metabolism interference
Source: J Biomed Sci. 2022 Feb 23;29:16. doi: 10.1186/s12929-022-00797-z (PMC8864857; doi:10.1186/s12929-022-00797-z)

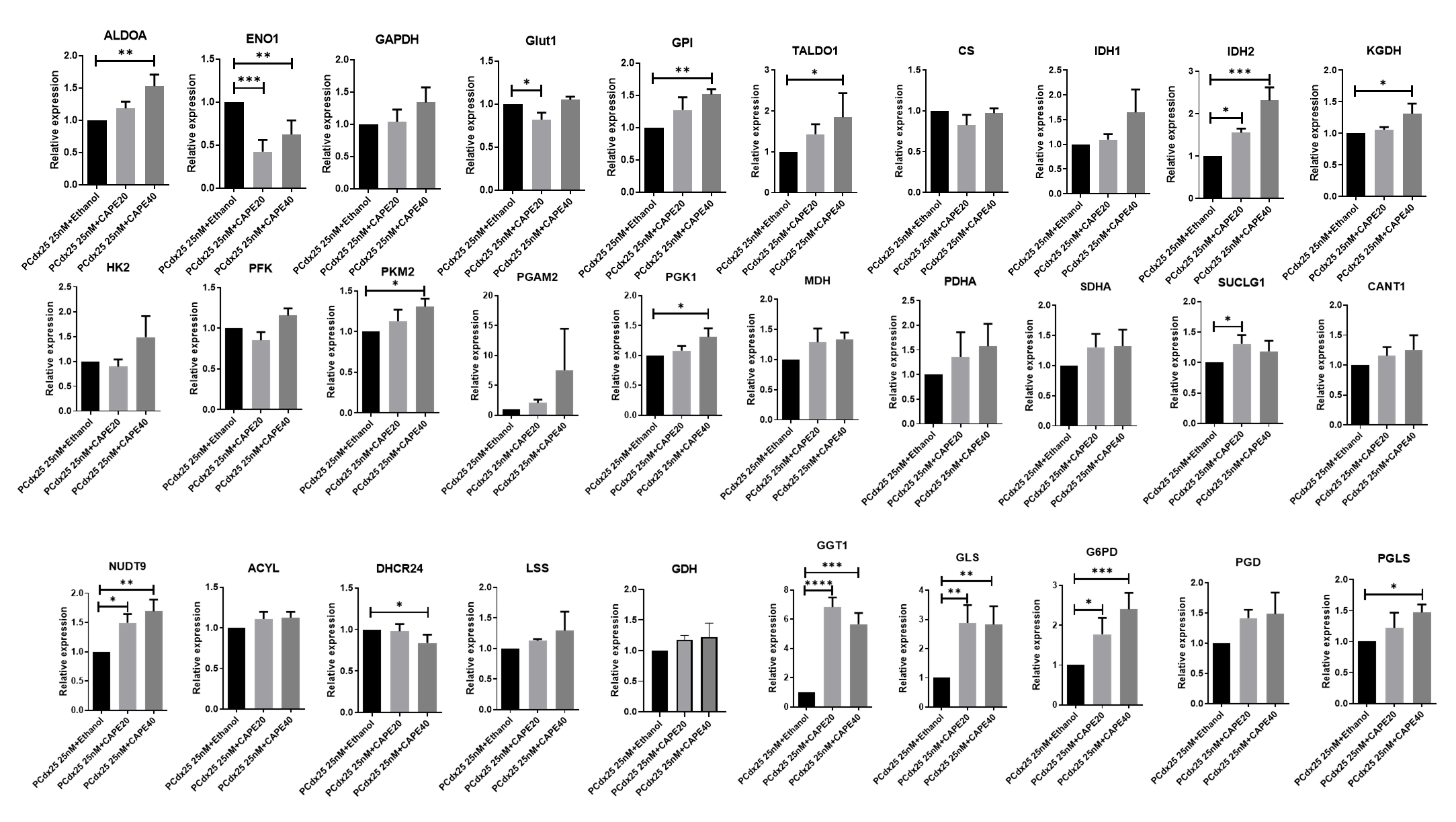

Supplement: Supplementary file 2 — Additional file 2: Figure S1. Effects of combination treatment on metabolic genes in PC/DX25 cells. Effects of combination treatment on metabolic genes in PC/DX25 cells treated with 25 nM docetaxel plus increasing concentration of CAPE (0, 10, 20, 40 μM) for 48 h were examined by qRT-PCR. [file 12929_2022_797_MOESM2_ESM.tif]

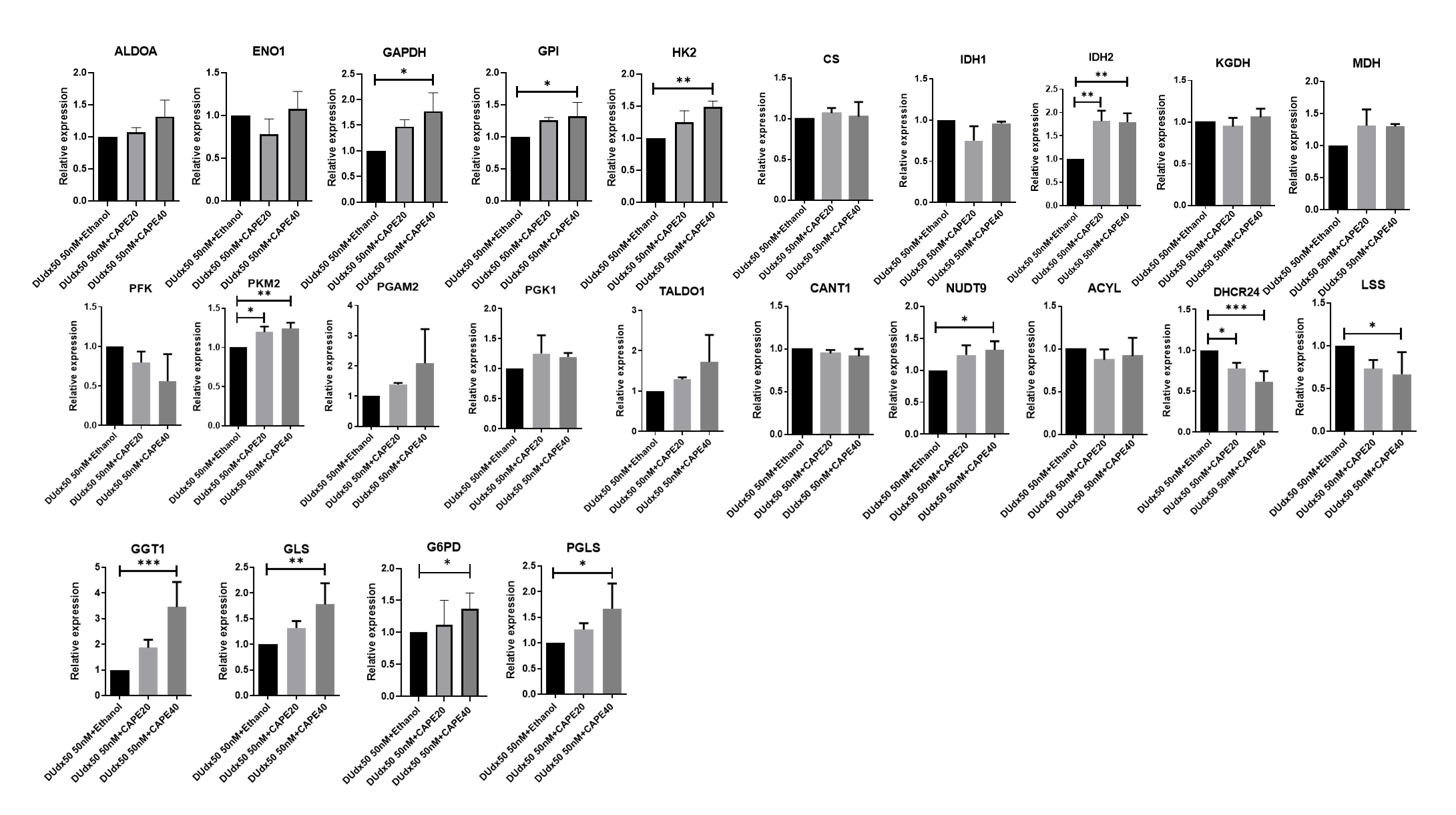

Supplement: Supplementary file 3 — Additional file 3: Figure SS. Effects of combination treatment on metabolic genes in PC/DX25 cells. Effects of combination treatment on metabolic genes in DU/DX50 cells treated with 50 nM docetaxel plus increasing concentration of CAPE (0, 10, 20, 40 μM) for 48 h were examined by qRT-PCR. [file 12929_2022_797_MOESM3_ESM.tif]
